# Soil quality indicator-based land productivity modelling for agricultural sustainability

**DOI:** 10.1371/journal.pone.0316840

**Published:** 2025-01-15

**Authors:** Ibraheem A. H. Yousif, Ali Abdel Hamid, Abdalsamad A. Aldabaa, Sayed A. Hassanein, Nazih Y. Rebouh, Elsayed Said Mohamed, Mohamed S. Shokr

**Affiliations:** 1 Soil Science Department, Faculty of Agriculture, Cairo University, Giza, Egypt; 2 Pedology Department, Water Resources and Desert Soils Division, Desert Research Center, Egypt; 3 Department of Environmental Management, Institute of Environmental Engineering (RUDN University), Moscow, Russia; 4 National Authority for Remote Sensing and Space Sciences, Cairo, Egypt; 5 Soil and Water Department, Faculty of Agriculture, Tanta University, Tanta, Egypt; Wroclaw University of Environmental and Life Sciences, POLAND

## Abstract

Rapid population expansion has made food security a global concern for humanity, necessitating a sustainable assessment of natural resources. Well evaluated and managed soil is one of the most significant resources that can assist close the gap between supply and demand for food to attain food security. A precise assessment of land productivity (LP) is essential for sustainable land use management. The primary aim of this research is to predict the land productivity index (LPI) for certain locations on Egypt’s northwest coast under current conditions using soil quality indicators (SQIs). Additionally, the study proposed relevant management actions to boost land productivity in the future. To achieve this aim, a spatial model was created to assess LPI for some areas in the north western coast of Egypt. LP equation was used to estimate current land productivity index (CLPI) and potential land productivity index (PLPI). Results found that the study area mainly located under two classes of CLPI, e.g., average (class ⅡⅠ) represents an area of 13322.63 ha (67.55%) and good grade (class Ⅱ) covers an area of 4339.78 ha (22%). PLPI grades were good (II) and average (III) with an area of 16192.54 ha (82.10%) and 1903.23 ha (9.65%), respectively. The finding showed that more than 60% of the studied area was converted from poor and average class to good class after applying the proposed solutions of soil improvement. The methodology presented here can easily be replicated under similar circumstances in arid zones, allowing local governments and decision-makers to utilize the quantitative results obtained to ensure sustainable development.

## 1. Introduction

Hunger and poverty constitute substantial global challenges for humankind, affecting more than 800 million people worldwide [1]. The African continent bears the greatest weight of these challenges, holding the most significant proportion on a global scale [2]. The economy of Egypt is predominantly dependent on agricultural activities, which concurrently supplies the principal portion of its food security [3]. Arid lands, like Egypt, which include dry sub-humid, semi-arid, arid, and hyper-arid conditions, are found in areas where the aridity index is less than 0.65. However, the sustainability of crop production is negatively impacted by the severe climate and scarce land resources that dry land agro-ecosystems encounter [4, 5]. To accomplish the sustainable crop production, it is necessary to pay attention to soil functions and ecosystem services. Therefore, it is essential to carry out comprehensive assessment of soil quality indicators (SQIs) and land productivity index (LPI) ongoing monitoring of land attributes in order to ensure optimal land use planning and the conservation of arable land resources [6–8]. The soil quality (SQ) concept is characterized as the capacity of soil to provide plants with vital nutrients throughout their growth phases, which is crucial for sustaining agricultural productivity [4, 9, 10]. Therefore, it is essential for long-term land resource management to assess environmental sustainability through SQIs [11]. A quantitative assessment of the physical, chemical, and biological attributes of soil serve as critical indicators of soil quality that can quickly respond to alterations in soil conditions [4, 11–15]. The SQIs indicate the geographical variations in the chemical and physical characteristics of soil, providing an extensive evaluation of soil productivity conditions [16]. Recently, the idea of SQIs has been introduced to handle these issues and offer a comprehensive evaluation of SQ [17–20] and many researches have been used this index in northwestern cost and many areas in Egypt to determine SQ [21–29].

LPI encompasses the aggregate output associated with a various factors, such as climatic conditions, morphological features of the terrain, and the physical and chemical characteristics of the soil [30, 31]. Assessment and monitoring the LP play a crucial role in enhancing the agricultural methodologies to preserve soil viability for producing food, fibers, and vital goods [32, 33]. Evaluating LP is usually done either directly or indirectly. Direct techniques use controlled management procedures and field, greenhouse, or laboratory research conducted under specific climate conditions. Indirect approaches rely on creating and utilizing models in order to calculate the LPI [34, 35]. One well-known approach to assessing LP is the parametric model that Riquier [36] presented. The LPI, which is produced by multiplying soil parameters connected to plant development, is a single numerical indicator that it gives [37]. Worldwide and in Egypt, Require technique has been utilized for measure and monitoring LP [38–45]. LPI is a useful tool for monitoring land productivity. This index correlates strongly with yield, thus it can actually explain yield variations [46, 47].

Traditional methods of soil quality assessment often rely on point-based sampling, which can be time-consuming, labor-intensive, and may not capture the spatial variability of soil properties across large areas. This study is considered an attempt to develop a new methodology for calculating land productivity index based on soil quality indicators in integration with the Riquier equation [36]. Given the conditions of the study area in terms of topography and rain-fed irrigation, soil depth and slope were considered as independent factors in the suggested equation. Since the investigated area is mainly characterized by the cultivation of figs and olives trees on rain-fed agriculture, the soil depth is very important for the growth of these trees [48, 49]. Furthermore, the importance of slope is its role in collecting rainwater needed to irrigate these trees [13, 49]. The spatial model-builder can assist in reducing the data set of soil analyzes while also lowering the operating costs of LP evaluation process [6, 28, 50, 51]. Consequently, the evaluation LP using geographic information systems GIS and remote sensing (RS) methodologies is very important for land evaluation, enabling reclamation and agricultural strategies, but this methodology needs to be applied in several of environments, especially arid and semi-arid areas. Whereas this research study can be used as an evaluating tool to assist decision-makers and land managers to map and evaluate LPI under arid and semi-arid environments. Egypt’s northwest coast is a region of strategic importance, both economically and environmentally [49, 52, 53]. This area, characterized by its Mediterranean climate, exhibits a diverse range of soil types influenced by various factors such as topography, climate, and human activities [24, 48, 54, 55]. The region’s soils vary from sandy and calcareous soils near the coast to more fertile alluvial soils further inland [14, 48, 49]. However, soil degradation due to overgrazing, unsustainable agricultural practices, and urbanization poses a significant threat to soil productivity [52, 56]. Addressing these challenges requires an integrated approach to LP assessment that considers both natural and anthropogenic factors. The current study’s concept can be applied to other locations of similar subject. Based on this meaning, the main objective of this study is to provide a new indicator of land productivity assessment that is suitable for application in rain-fed agriculture environments in dry zones, by applying and adapting current concepts for assessing land productivity to the conditions of the northwestern coast of Egypt. Specifically, the study aims at evaluating and modeling the current land productivity index (CLPI) and potential land productivity index (PLPI) based on soil quality indicators at some rain-fed areas of north western cost of Egypt to support regional and global efforts in sustainable agriculture.

## 2. Materials and methods

### 2.1 The area of study

**Fig 1:**
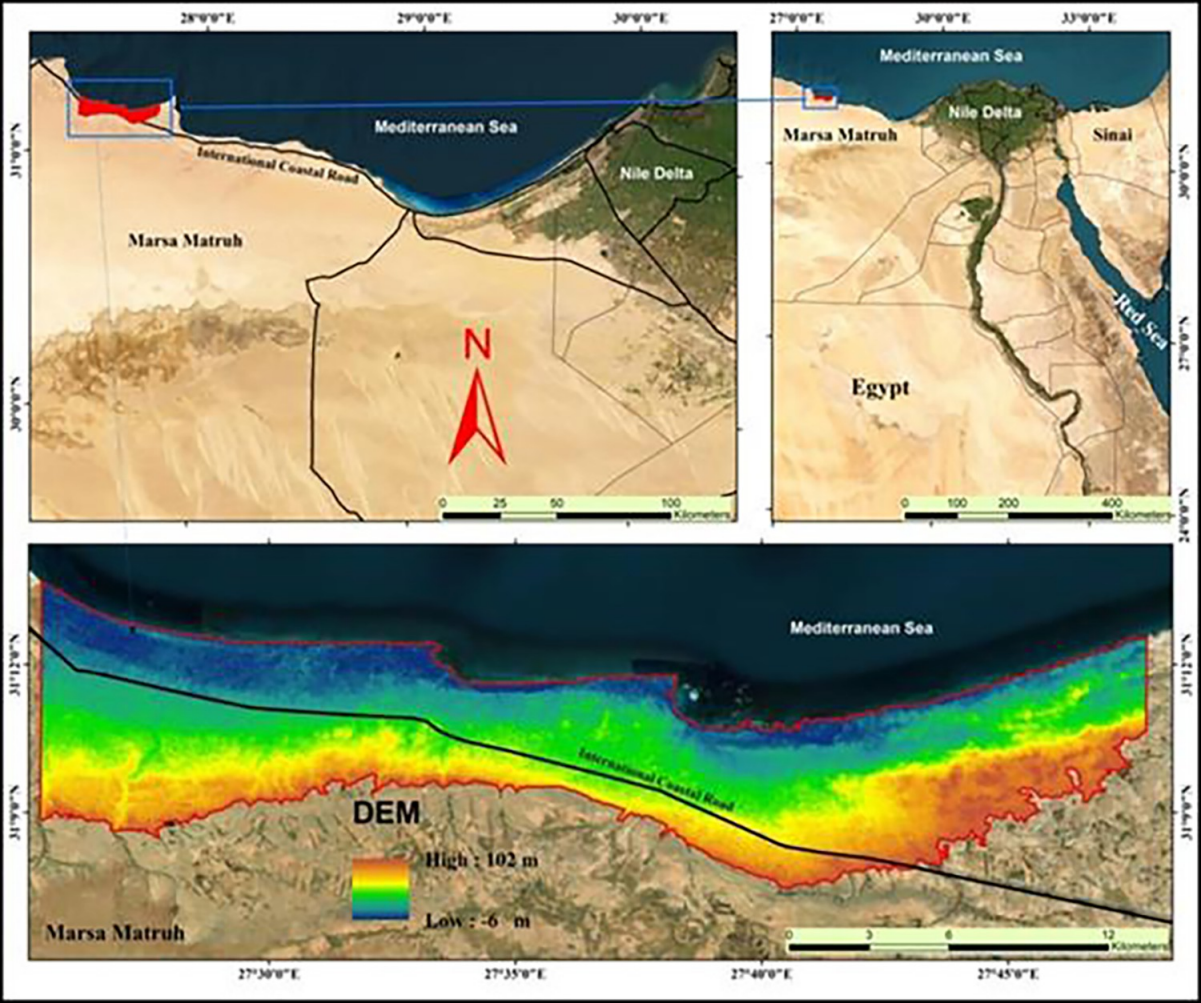
Location of the study area.

**Fig 2:**
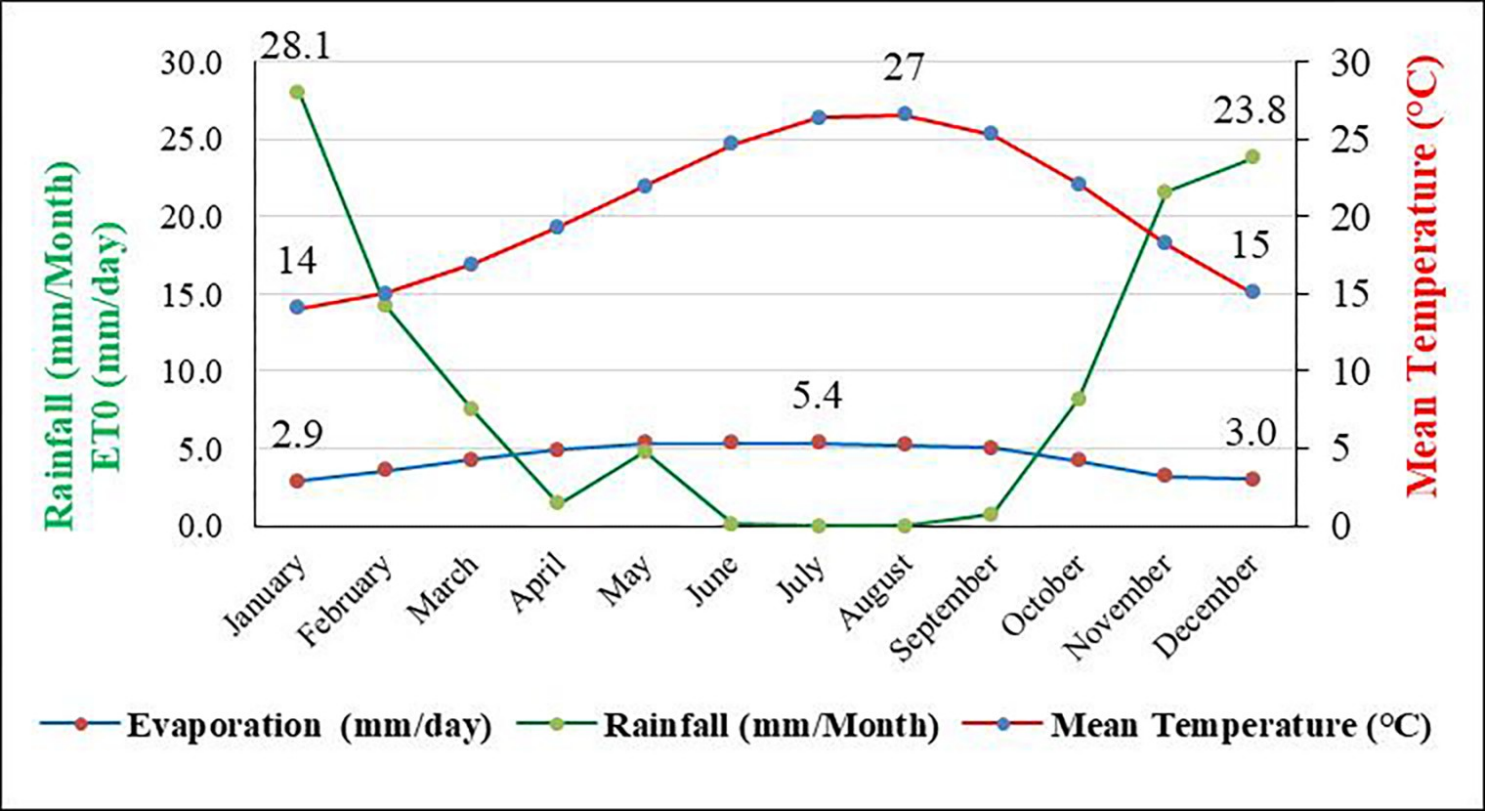
Climatological data of the investigated area (2005 to 2020) referred to Matrouh station–Egypt.

The study area is located at the east of Matrouh city by about 25 km. The area under investigation occupies an area of 197.22 km^**2**^ (19721.87 hectare) and existed between longitudes 22° 25’ 25” to 27° 47’ 51" E and latitudes 31° 7’ 27" to 31° 13’ 42" N (Fig 1) The satellite image was downloaded from(**https://earthexplorer.usgs.gov**). Since the study was carried out on publically accessible property and did not include interactions with controlled species or habitats, field site access and research activities were exempt from the need for special permits. The elevation of the studied area ranged between 11–120 m ASL as shown in the DEM (Fig 1). According to FAO [57], flat to nearly level, gently sloping and sloping are the dominant slope classes in the investigated area. The main agriculture land use types are scattered orchard trees like fig and olive, which are planted in the Wadi channels and Deltas. Barely planted in flat to almost flat soil on Marmarica plateau is also one of the main agricultural land use types. Furthermore, some scattered patches in this area are cultivated by wheat crops and some vegetables are suitable for the investigated area conditions. The climate of the investigated area is characterized by dry hot summer where the mean monthly temperature is ranged between 14.5 to 27°C, and almost rainy winter where the annual rainfall ranged between 87.10 and 274.50 mm year^**-1**^ with an average of 145.06 mm year^**-1**^, mean wind speed is 18.8 km hr^**-1**^, and the relative humidity ranged between 56 to 66% [58]. According to Soil Survey Staff [59], torric moisture and hyper-thermic temperature are the common regimes in the investigated area (Fig 2). The investigated area is dominated by a sedimentary rock varying from Tertiary (Miocene) to Quaternary period. The Miocene formation distributed in the tableland while the Quaternary formations (Pleistocene and Holocene) are distributed in the coastal plain, [60].

### 2.2 Geomorphology

**Fig 3:**
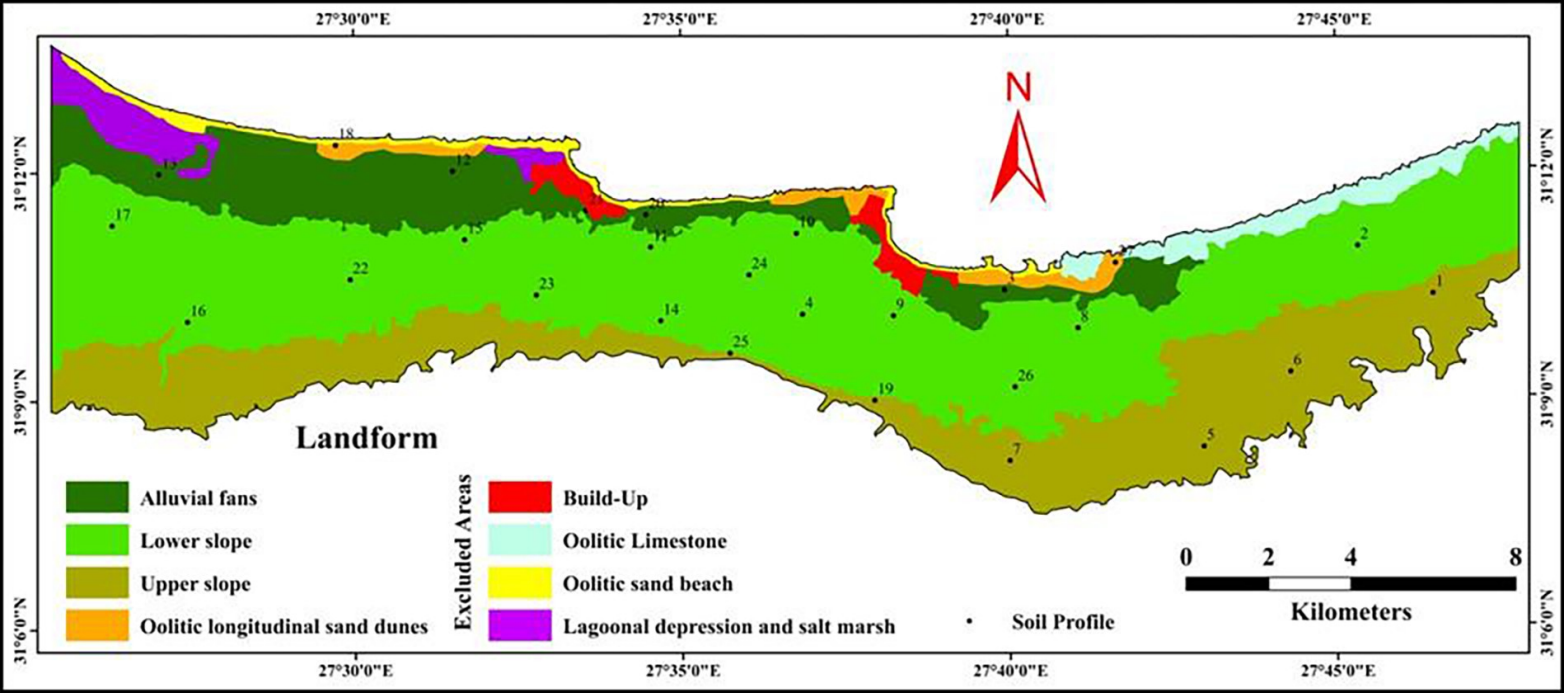
Geomorphology map and location of the represented soil profiles.

The main landforms in the area are upper slope (US), lower slope (LS), alluvial fans (AF) and oolitic longitudinal sand dunes (OLSD). The other units are excluded areas which are lagoonal depression and salt marsh, oolitic Limestone, oolitic sand beach, and build-up area (Fig 3). The geomorphological map has been modified based on Youssef et al., [49]. This map was produced using digital processing of Landsat ETM+ satellite images and digital elevation model. The map was imported to Arc GIS 10.8 [61], georeferenced and screen-on digitizing was performed to delineate different units to be used as a base for maps production.

### 2.3 Field and lab. work

Twenty-seven soil profiles represented the various geomorphological units in the examined area were dug and morphologically described according to FAO [57]. The exact locations of the profiles (Fig 3) were identified in the field using the Global Positioning System. The laboratory analyses were performed following the standard methods of Soil Survey Staff [62]. The investigated soils were classified according to Soil Survey Staff [59] Salinity (electric conductivity), soil acidity (pH) in saturated paste, cation exchange capacity (CEC), were measured in accordance with [63–65]. Soil organic matter content was measured using the acid-dichromate potassium and titration method [66]. Kjeldahl method was used to calculate soil nitrogen (N). A flame photometer was used to measure potassium (K) in the soil, and a spectrophotometer was used to measure phosphorus (P) in accordance with [67]. Flame photometer was used to quantify the available potassium concentrations in the soil [68]. The formula given by van Reeuwijk [69] was used to calculate the exchangeable sodium percentage (ESP). The percentages of sand, silt, and clay in the soil were analyzed using an international pipette method, and the international texture triangle was used for determining the texture of the soil [69].

### 2.4 Statistical analysis and spatial variability mapping

All obtained data from laboratory analysis were calculated as weighted mean value for each soil property according to Eq (1).


$$V=\sum_{i=1}^{n}\frac{\left(vi*ti\right)}{T}$$ Eq (1)

Where: (***V***) is soil property; (***vi***) is soil property value for each layer; (***ti***) is the thickness of each layer; (***T***) is the total depth of soil profile.

Descriptive statistical data was done using SPSS software V28. ArcGIS 10.8 software (ESRI, 2022) was used for mapping the spatial variability of the outputs of the research work. The spatial variability maps of the land capability classes in the research area were produced using the Inverse Distance Weighing (IDW) interpolation method. In addition, maps of soil productivity and soil quality index were made using it. "Inverse distance weighting" and "kriging" are two interpolation methods that are frequently employed in agriculture [70, 71]. Both approaches use measurements from nearby locations, assigning weights to individual measurements, to estimate values at unsampled places. While "kriging" requires more effort and time to complete, "inverse distance weighting" is simpler to use [72].

### 2.5 Soil quality indicators (SQIs)

In this study, twenty seven soil profiles were employed to assess SQIs, which included six physical, six chemical, and three fertility indicators, as shown in Table 1. These criteria were chosen because of their importance in soil quality and crop production. All of these indicators were assessed in soil samples from each profile to establish SQIs and assess the impact of cultivation management on soil function in the profiles’ locations [73]. Each index includes some soil properties or indicators, each of them was rated using the corresponding score from the Table 1. Afterwards, each soil quality index was calculated using the suitable formula (Eqs 2, 3, and 4).

**Table 1:** Rating of soil properties in each soil index (chemical, physical, and fertility).

Indicator	Description	Threshold	Score	Reference
Depth (cm)	Very deep	> 150	1	[57]
	Deep	100–150	0.8	
	Moderately deep	50–100	0.6	
	Shallow	30–50	0.4	
	Very shallow	< 30	0.2	
Slope (%)	Very gently sloping	< 2	0.8	
	Gently sloping	2–5	1	
	Sloping	5–10	1	
	Strongly sloping	10–15	0.5	
	Moderately steep	15–30	0.4	
	Steep	30–60	0.2	
	Very steep	> 60	0.2	
**Soil Chemical Index (SCI) = [pH × ECe × CEC × ESP × OM × CaCO3 × gypsum]**^**1/7**^ ……**Eq 2**				
pH (Soil Reaction)	Neutral	6.5–7.3	1	[74]
	Slightly alkaline	7.3–7.8	0.8	
	Moderately alkaline	7.8–8.4	0.6	
	Strongly alkaline	8.4–9.0	0.4	
	Very strongly alkaline	> 9	0.2	
ECe dS m^-1^ (Electrical Conductivity)	Non-saline	< 2	1	
	Very slightly saline	2–4	0.8	
	Slightly saline	4–8	0.6	
	Moderately saline	8–16	0.4	
	Strongly saline	> 16	0.2	
CEC (Cation Exchange Capacity)	Very high	> 40	1	[75]
	High	25–40	0.8	
	Moderate	12–25	0.6	
	Low	6–12	0.4	
	Very low	< 6	0.2	
ESP (Exchangeable Sodium Percent)	Non- sodic	< 10	1	[76]
	Slightly sodic	10–15	0.8	
	Moderately sodic	15–30	0.6	
	Strongly sodic	30–50	0.4	
	Very strongly sodic	> 50	0.2	
OM (g kg^-1^) Organic matter	Very high	> 50	1	[75]
	High	50–30	0.8	
	Moderate	30–17	0.6	
	Low	17–10	0.4	
	Very low	< 10	0.2	
CaCO_3_ g kg^-1^ (Calcium Carbonate)	Non-calcareous	0	1	[77]
	Slightly calcareous	0–20	1	
	Moderately calcareous	20–100	1	
	Strongly calcareous	100–250	1	
	Extremely calcareous	> 250	1	
**Soil Physical Index = [Gravel × Texture × BD × WHC × HC]**^**1/5**^ ……………………..…… **Eq 3**				
Gravel (%)	Very high	> 75	0	
	High	55–75	0.4	
	Moderate	35–55	0.6	[77]
	Low	15–35	0.8	
	Very low	0–15	1	
Texture	Clay loam	---	1	[75]
	Silty clay loam, loam, silty clay, silt	---	0.9	
	Silt loam, clay < 60%	---	0.8	
	Sandy clay, sandy clay loam, sandy loam	---	0.7	
	Clay > 60%	---	0.6	
	Loamy sand	---	0.4	
	Sand	---	0.2	
BD (g cm^3^) Bulk Density	Very low	<1.2	1	
	Low	1.2–1.4	0.8	
	Moderate	1.4–1.6	0.6	
	High	1.6–1.8	0.4	
	Very high	> 1.8	0.2	
WHC (%) Water Holding Capacity	Very high	> 35	1	
	High	35–30	0.8	
	Moderate	30–20	0.6	
	Low	20–15	0.4	
	Very low	< 15	0.2	
HC (%) Hydrolytic Conductivity	Low	1.0–2.0	1	
	Moderate	2.0–6.0	0.8	
	Very low	0.05–1.0	0.6	
	High	6.0–12.0	0.4	
	Extremely low—very high	< 0.05 or > 12.0	0.2	
**Soil Fertility Index (SFI) = [N × P × K]**^**1/3**^ ……………………………………………..………….. **Eq 4**				
AN (Mg kg^-1^) Available Nitrogen	Very high	> 120	1	[78]
	High	120–100	0.8	
	Moderate	100–75	0.6	
	Low	75–30	0.4	
	Very Low	< 30	0.2	
AP (Mg kg^-1^) Available Phosphorus	Soil texture	Light/Medium/Heavy	---	[79]
	Very high	> 15/> 8/> 5	1	
	High	15–10/8–5/5–3	0.7	
	Medium	10–5/5–3/3–2	0.5	
	Low	< 5/< 3/< 2	0.2	
AK (Mg kg^-1^) Available Potassium	Very high	> 180	1	
	High	180–120	0.7	
	Medium	120–60	0.5	
	Low	< 60	0.2	

### 2.6 Current and potential land productivity assessment

Evaluation of land productivity is a multidisciplinary approach involving various methods, system and factors. In this study, a new equation was proposed to evaluate the current land productivity index (CLPI) based on soil quality indicators to match the conditions of the study area (Eq 5).


$$LPI=[Depth*Slope*[SCI*SPI*SFI{]}^{1/3}]*100$$ Eq (5)

Where: LPI = Land Productivity Index; SCI = Soil Chemical Index; SPI = Soil Physical Index; SFI = Soil Fertility Index.

Whereas the investigated area is located on the Northwestern Mediterranean coast of Egypt and depends mainly on rain-fed irrigation. Therefore, the slope gradient and soil depth are considered the most important factors determining the productivity in the study area. In other words, the importance of the slope is evident in its role in collecting rainwater, which leads to saturate the soil profile with the required irrigation water for crop growth and productivity. In this sense, the slope and soil depth were placed as independent factors in the land productivity equation.

This equation allowed the calculation of the current and potential productivity. It involves calculating a land productivity index based on five factors and primarily concerned with intrinsic soil characteristics that control soil utilization and productive capacity (Eq 5). Each factor is given a numeric value from 0 to 100. The resultant index obtained by a multiplication of those factors is positioned in a specific productivity classes (Table 2).

**Table 2:** Soil productivity classes.

Soil Productivity Index	Class	Symbol
70–100	Excellent	I
40–70	Good	II
25–40	Average	III
10–25	Poor	IV
0–10	Extremely Poor	V

After improving soil properties which considered as limitations of land productivity, the potential land productivity index (PLPI) of the estimated potential productivity could be determined using the same eruption with the new rating of soil properties.

### 2.7 Spatial model development for assessment current and potential land productivity index

**Fig 4:**
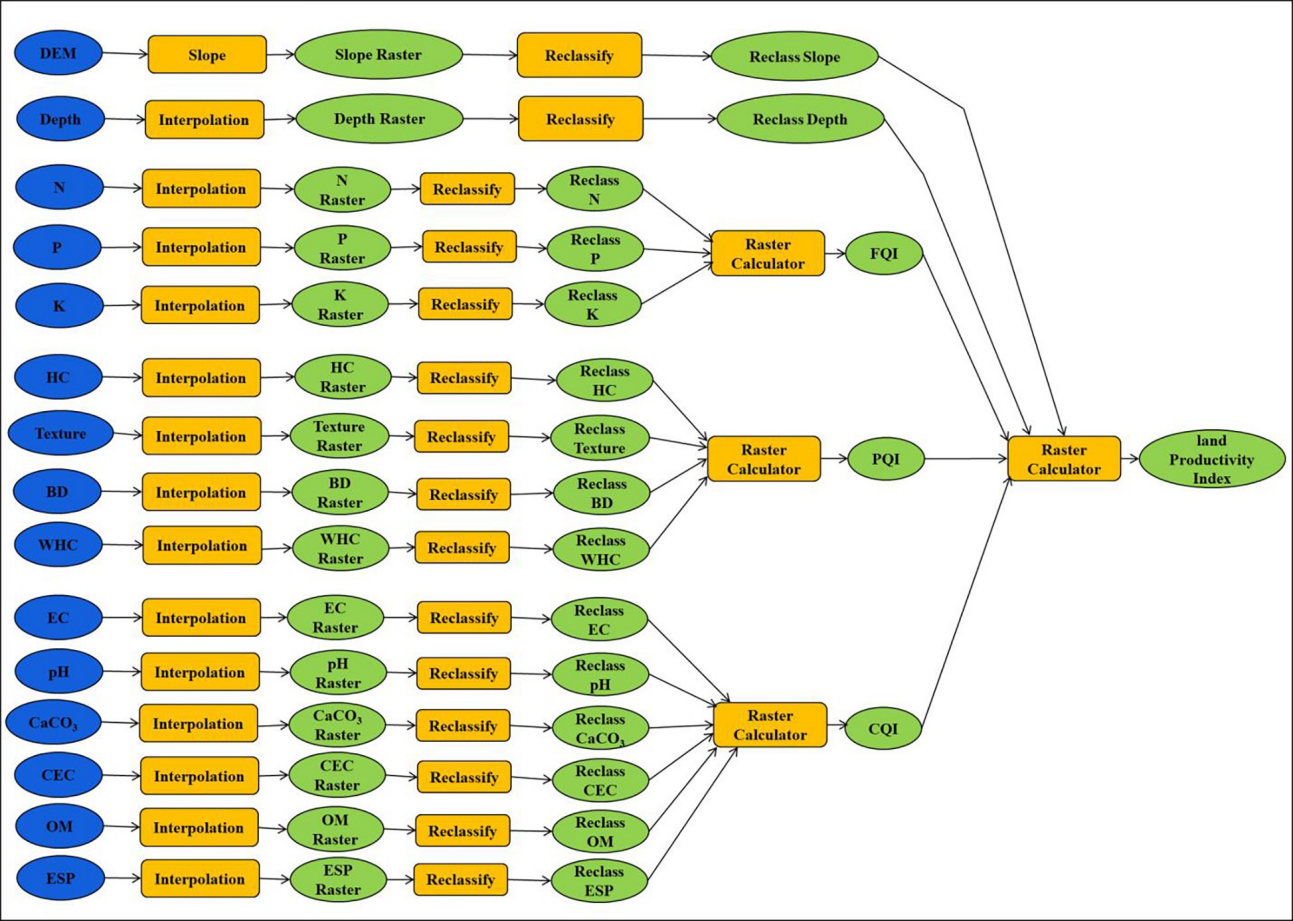
Spatial model structure for assessing land productivity index.

The Model Builder tool in ArcGIS was used to create the spatial model for LPI (Fig 4). Model Builder was used to automate some spatial analysis and data management processes. It also generated a diagram showing the linked chains of geo-processing tools, where the output of one process is used as the input for another. To determine each soil indicator’s weighting factor and produce the final CLPI and PLPI maps (Fig 4), the following approaches were used in this study: First, different soil properties interpolated from point-based to a raster layer, second, different soil properties raster layers reclassified according to Table 1, third, different class of soil raster layers assigned to an LPI index (Table 7). Fourth, feeding Eq 5 by different outputs from the previous step to map LPI. Finally, reclassify the resulted raster layer from the previous step to get the final land productivity map.

## 3. Results

### 3.1 Physiographic of the investigated area

The physiographic units of the studied area are represented in Fig 3 and Table 3. The Lower slope unit (LS) covers an area of 9979.90 ha representing 50.60% out of the investigated area. While, The upper slope unit (US) cover an area of 5176.78 ha representing 26.25% out of the studied area. The Alluvial fans (AF) and Oolitic longitudinal sand dunes (OLSD) units occupy an area of 2607.30 and 331.78 ha representing 13.22 and 1.68% out of the studied area, respectively.

**Table 3:** Legend of the physiographic soil map of the study area.

Landform	Area hectare	%	Represented Profiles	Soil Taxonomy
Upper slope (US)	5176.78	26.25	23	*Typic Torripsamments*
			5, 16, 25	*Typic Torriorthents*
			1, 19	*Typic Haplocalcids*
			6, 7	*Lithic Torriorthents*
Lower slope (LS)	9979.90	50.60	2, 4, 8, 14, 15, 24	*Typic Haplocalcids*
			9, 10, 11, 26	*Typic Torriorthents*
			17	*Lithic Torriorthents*
			22	*Calcic Haplosalids*
Alluvial fans (AF)	2607.30	13.22	21	*Typic Haplosalids*
			3, 12	*Typic Torriorthents*
			13, 20	*Typic Haplocalcids*
Oolitic longitudinal sand dunes (OLSD)	331.78	1.68	18, 27	*Typic Torripsamments*
Build-up	263.57	1.34	Excluded Areas	
Lagoonal depression and salt marsh	469.96	2.38		
Oolitic Limestone	518.23	2.63		
Oolitic sand beach	374.47	1.90		
Total	19722	100		

### 3.2 Soil classification

The soil profiles were classified as demonstrated in Table 3. From the obtained data, the studied soil profiles were found to be within two soil orders (Entisols and Aridisols). Entisols order was dominant, covering around 55.5% of the studied area (15 soil profiles), and it was classified into three subgroups *Typic Torriorthents* and *Typic Torripsamments*, and *Lithic Torriorthents*. While Aridisols order covered about 44.5% of the total soil profiles (12 soil profiles), it was classified into one subgroup *Typic Haplocalcids*, *Typic Haplosalids*, and *Calcic Haplosalids*. Table 4 shows physical, chemical, and fertility analyses of the representative soil profiles.

**Table 4:** Soil physical and chemical properties of the investigated soils.

Unit	No.	Depth	Gravel	Sand	silt	clay	Tex.	BD	WHC	HC	EC	pH	CaCO_3_	OM	ESP	CEC	N	P	K
US	1	120	0.93	80.71	8.75	10.54	LS	1.58	27.50	2.65	1.88	7.06	84.33	0.45	10.14	6.99	56.33	0.84	93.60
	5	120	0.48	70.92	11.04	18.04	SL	1.48	37.79	0.87	3.05	7.81	34.93	0.76	10.70	11.90	76.83	2.22	92.51
	6	50	20.78	69.88	13.75	16.37	SL	1.49	38.95	1.09	6.06	7.34	43.51	0.57	12.13	10.37	64.46	1.42	101.79
	7	65	0.27	78.53	10.00	11.47	SL	1.56	30.02	2.25	2.01	7.51	25.16	0.51	10.20	7.67	60.20	1.14	93.00
	16	120	18.23	71.55	10.83	17.62	SL	1.49	37.14	0.92	0.88	7.26	49.44	1.04	4.40	12.75	95.46	2.17	91.34
	19	130	9.25	80.45	9.51	10.04	SL	1.59	27.87	2.87	3.13	7.23	23.89	0.63	10.74	7.42	68.26	3.48	92.16
	23	150	0.06	83.50	10.50	6.00	LS	1.67	24.54	5.87	7.11	7.95	20.62	0.26	12.64	4.00	43.69	0.36	95.35
	25	150	19.23	77.67	7.42	14.92	SL	1.52	30.83	1.33	3.39	7.87	22.77	0.44	10.86	9.13	55.55	1.07	90.29
LS	2	115	0.48	68.14	11.63	20.23	SL	1.46	40.47	0.67	3.11	7.18	55.49	0.22	10.73	10.94	40.52	0.24	95.15
	4	40	0.27	75.71	10.00	14.29	SL	1.53	32.86	1.46	5.45	7.45	24.65	0.41	11.85	8.70	53.51	1.07	106.23
	8	80	0.77	67.54	11.56	20.90	SL	1.45	41.07	0.62	3.98	7.34	55.09	0.35	11.14	11.77	49.33	0.52	95.65
	9	105	0.10	71.55	11.19	17.26	SL	1.49	37.21	0.96	1.12	7.76	39.65	0.98	9.77	12.36	91.77	1.66	94.01
	10	75	20.78	76.88	7.00	16.12	LS	1.51	31.56	1.10	0.75	7.47	82.61	0.46	9.60	9.80	56.77	0.86	91.05
	11	120	2.12	70.71	11.67	17.62	SL	1.49	38.08	0.92	4.93	7.74	43.80	0.61	11.60	11.12	66.67	1.26	98.22
	14	105	0.05	76.78	8.10	15.12	SL	1.52	31.48	1.27	4.52	7.74	34.93	0.47	11.40	9.35	57.56	1.58	96.55
	15	135	0.15	82.66	6.30	11.05	LS	1.58	25.46	2.47	0.61	7.60	57.66	0.65	9.53	8.00	69.73	2.11	90.93
	17	40	7.90	71.76	10.00	18.25	SL	1.48	36.86	0.84	1.51	7.59	36.32	0.52	9.96	11.09	60.72	1.63	94.87
	22	115	0.00	49.94	38.24	11.82	L	1.50	54.85	2.11	34.31	8.12	23.23	0.58	25.64	8.10	64.60	1.77	97.34
	24	70	1.28	83.96	8.75	7.29	LS	1.64	24.03	4.73	7.37	8.15	41.16	0.59	12.76	5.88	65.39	2.48	97.52
	26	150	7.27	72.50	8.33	19.17	SL	1.48	36.05	0.75	10.18	7.72	33.42	0.21	14.10	10.37	39.85	0.24	90.41
AF	3	130	0.27	79.30	4.04	16.66	SCL	1.51	28.52	1.03	3.69	7.82	68.27	0.61	11.00	10.63	66.56	1.09	95.80
	12	150	0.05	68.05	14.50	17.45	SL	1.48	40.75	0.95	12.52	7.49	36.26	0.94	15.22	12.31	89.11	6.49	126.05
	13	130	0.15	67.38	8.08	24.54	SCL	1.43	40.91	0.41	1.33	7.89	37.48	1.08	9.87	16.39	98.65	2.56	94.18
	20	60	7.90	78.23	9.46	12.31	SCL	1.55	30.03	1.97	2.06	7.08	21.34	0.40	10.22	7.68	52.86	0.59	90.30
	21	150	3.06	70.76	16.95	12.28	SL	1.54	37.34	1.95	20.19	8.05	51.22	0.62	18.89	8.50	67.65	1.93	107.47
OLSD	18	150	0.06	97.38	2.50	0.12	S	1.68	9.05	10.41	0.59	7.90	99.36	0.11	9.52	0.48	33.42	0.14	90.67
	27	150	19.23	99.09	0.79	0.12	S	1.69	11.87	13.71	0.46	8.18	97.36	0.24	20.27	0.98	42.30	0.60	89.78
Min		40	27.96	49.94	0.79	0.12	-	1.43	9.05	0.41	0.46	7.06	20.62	0.11	4.40	0.48	33.42	0.14	89.78
Max		150	0.00	99.09	38.24	24.54	-	1.69	54.85	13.71	34.31	8.18	99.36	1.08	25.64	16.39	98.65	6.49	126.05

LS = Lower slope; AF = Alluvial fans; US = Upper slope; OLSD = Oolitic longitudinal sand dunes; Tex = Texture Class; BD = Bulk Density; WHC = Water Holding Capacity; HC = Hydraulic Conductivity; EC = Electric Conductivity; pH = Soil reaction; CaCO3 = calcium carbonate; OM = Organic Matter; ESP = Exchangeable Sodium Percentage; CEC = Cation Exchange Capacity; N = Nitrogen; K = Potassium; P = Phosphorus

### 3.3 Descriptive statistics of soil properties

The descriptive statistical analysis of the laboratory soil profiles’ data is shown in Table 5. The obtained data on the soil’s physical properties revealed that soil depth in the studied soil profiles ranged from 40 to 150 cm with a mean value of 110.19 cm. Regarding the particle size distribution, the sand fraction was dominant in the examined soil profiles, whereas it varied from 49.94 to 99.09% with an average of 75.61%. The silt fraction was the second dominant fraction in the soil profiles; it ranged from 0.79 to 38.24% with an average value of 10.40%. The clay fraction was the lowest contributor with minimum, maximum, and mean values of 0.12, 24.54, and 13.99%, respectively. The hydraulic conductivity varied from 0.41 to 13.71 cm/hr, while the mean value was 2.45 cm/hr. Therefore, the infiltration rate of these studied soil profiles ranged from very low to high classes [75]. Concerning the water holding capacity varied between 9.05 and 54.85, while the mean value was 32.71%. The soil bulk density, values differed from 1.42 to 1.69 g/m^**3**^ with a mean value of 1.53 g/m^**3**^. Concerning to the soil’s chemical properties, the obtained data revealed that the organic matter content in the studied profiles ranged from 0.11 to 1.08%, with an average of 0.54%. While the values of calcium carbonate ranged from 20.62 to 99.36% with an average of 46.07%. The soil profiles varied from strongly to extremely calcareous soils [57]. According to the soil pH values in the studied soil profiles, the data varied between 7.06 and 8.18 (mean = 7.64). The ECe values of the studied soil profiles differed from 0.46 to 34.31 dS/m with an average of 5.41 dS/m. whilst the ESP values of the studied soil profiles ranged from 4.40 to 25.64%, with a mean value of 12.03%. Moreover, the CEC values in the examined soil profiles ranged from 0.48 to 16.39 cmol(+)/kg, while the mean value was 9.06 cmol+/kg. Regarding the soil fertility properties, the available nitrogen ranged between 33.42 to 98.65 mg/kg, while the mean value was 62.51 g/kg. The available phosphorus varied from 0.14 to 6.49 with an average of 1.54 mg/kg. According to the available potassium, the values differed between 89.78 and 126.05 mg/kg (mean = 96.01 mg/kg).

**Table 5:** Descriptive statistics of the investigated soils.

Property	Unit	Range	Min	Max	Mean	Std. D	Variance	CV %	Skewness	Kurtosis
EC	dSm^-1^	33.85	0.46	34.31	5.41	7.23	52.26	133.52	2.96	9.96
pH		1.12	7.06	8.18	7.64	0.33	0.11	4.29	-0.13	-0.94
CaCO_3_	%	78.74	20.62	99.36	46.07	22.84	521.80	49.58	1.09	0.41
OM	%	0.97	0.11	1.08	0.54	0.25	0.06	46.27	0.55	0.00
ESP		21.24	4.40	25.64	12.03	4.05	16.36	33.62	1.81	4.74
CEC	cmolc (+)/ kg	15.91	0.48	16.39	9.06	3.46	11.94	38.14	-0.76	1.36
N	mg/kg	65.23	33.42	98.65	62.51	16.90	285.72	27.04	0.55	0.02
P	mg/kg	6.35	0.14	6.49	1.54	1.29	1.65	83.64	2.32	7.82
K	mg/kg	36.27	89.78	126.05	96.01	7.52	56.50	7.83	2.78	9.55
Depth	cm	110.00	40.00	150	110.19	36.78	1352.85	33.38	-0.65	-0.85
gravel	%	27.96	0.00	27.96	6.51	8.47	71.67	130.02	1.17	0.08
Sand	%	49.15	49.94	99.09	75.61	9.52	90.66	12.59	0.26	2.48
Silt	%	37.45	0.79	38.24	10.40	6.52	42.57	62.71	3.01	13.13
Clay	%	24.42	0.12	24.54	13.99	5.80	33.63	41.47	-0.86	0.85
BD	gcm^-1^	0.26	1.43	1.69	1.53	0.07	0.00	4.60	0.98	0.18
WHC	%	45.80	9.05	54.85	32.71	9.23	85.12	28.21	-0.55	1.83
HC	mmhr^-1^	13.30	0.41	13.71	2.45	3.07	9.42	125.24	2.76	7.72

Tex = Texture Class; BD = Bulk Density; WHC = Water Holding Capacity; HC = Hydraulic Conductivity; EC = Electric Conductivity; pH = Soil reaction; CaCO3 = calcium carbonate; OM = Organic Matter; ESP = Exchangeable Sodium Percentage; CEC = Cation Exchange Capacity; N = Nitrogen; K = Potassium; P = Phosphorus; Min = Minimum; Max = Maximum; Std. D = Stander Deviation; CV = Coefficient of Variance.

The results show that soil depth (36.78) and CaCO3 (22.84) have the highest values of Std. D which indicate significant variability. Whereas soil pH (0.33) and OM (0.25) have the lowest values of Std. D which for reflect more uniform distributions. The findings illustrate that soil pH (4.29%) and BD (4.60%) have the lowest CV values, while EC (133.52%) and HC (125.24%) show extreme variability with the highest values of CV. Regarding to Skewness, results indicate that EC (2.96), ESP (1.81), and P (2.32) have a positive skewness indicates longer tails to the right, with a concentration of lower values and a few high outliers. While CEC (-0.76) and WHC (-0.55) have a negative skewness suggests longer tails to the left, with higher values more common. The results of Kurtosis explain that EC (9.96), silt (13.13), and P (7.82) have high kurtosis values which indicate peaked distributions with extreme values, while soil pH (-0.94) and OM (0.00) have the lowest values of kurtosis which reflects flatter distributions.

### 3.4 Correlation analysis among soil quality properties

Data of correlation coefficients of all soil properties was shown in Table 6. From the obtained data, it was clear that high correlation was recorded between some of soil parameters. For instance, ESP was highly positive correlated with EC and pH with correlation coefficients r = 0.815 and 0.553, respectively. CEC has a strong positively correlated with OM (r = 0.675) and low correlated with other parameters. Sand was highly positive correlated with BD (r = 0.824) and HC (r = 0.774), while highly negative with silt (r = -0.802), clay (r = -0.740), and WHC (r = -0.995). Clay was highly positive correlated with WHC (r = 0.776) and highly negative with BD (r = -0.969) and HC (r = -0.885). Total nitrogen was in high positive correlation with organic matter and CEC. Available P has a high positive correlation with organic matter and N. Available K was in high positive correlation with EC and available P. However, these data could be useful to distinguish the soil parameters’ relation.

**Table 6:** Correlation coefficients of soil properties in the studied soil profiles.

	EC	pH	CaCO_3_	OM	ESP	CEC	N	P	K	depth	gravel	sand	silt	clay	BD	WHC	HC
EC	1																
pH	.381*	1															
CaCO_3_	-.312	.046	1														
OM	.035	-.008	-.269	1													
ESP	.815**	.553**	-.043	-.193	1												
CEC	-.008	-.284	-.377	.675**	-.300	1											
N	.032	-.008	-.268	1.0**	-.193	.672**	1										
P	.217	-.008	-.281	.704**	.085	.358	.705**	1									
K	.435*	.001	-.201	.300	.317	.234	.301	.669**	1								
depth	-.352	-.021	.381*	.056	-.244	-.181	.058	.077	-.110	1							
gravel	.072	-.058	.281	-.243	.268	-.058	-.241	-.256	.184	-.246	1						
sand	-.010	.137	.330	-.073	-.157	-.250	-.076	-.193	-.099	.191	-.057	1					
silt	-.036	-.182	-.087	.019	-.003	.109	.019	.111	.038	-.086	-.063	-.802**	1				
clay	.057	-.020	-.443*	.099	.260	.287	.102	.192	.120	-.217	.165	-.740**	.191	1			
BD	-.029	.098	.458*	-.090	-.206	-.262	-.094	-.203	-.104	.226	-.147	.824**	-.341	-.969**	1		
WHC	.022	-.128	-.331	.038	.185	.228	.040	.186	.119	-.203	.073	-.995**	.763**	.776**	-.851**	1	
HC	-.166	.040	.507**	-.080	-.329	-.289	-.082	-.183	-.192	.313	-.230	.774**	-.342	-.885**	.864**	-.787**	1

*. Correlation is significant at the 0.05 level (2-tailed).

**. Correlation is significant at the 0.01 level (2-tailed).

Tex = Texture Class; BD = Bulk Density; WHC = Water Holding Capacity; HC = Hydraulic Conductivity; EC = Electric Conductivity; pH = Soil reaction; CaCO3 = calcium carbonate; OM = Organic Matter; ESP = Exchangeable Sodium Percentage; CEC = Cation Exchange Capacity; N = Nitrogen; K = Potassium; P = Phosphorus

### 3.5 Soil quality indicators

#### 3.5.1 Soil chemical index (SCI)

**Fig 5:**
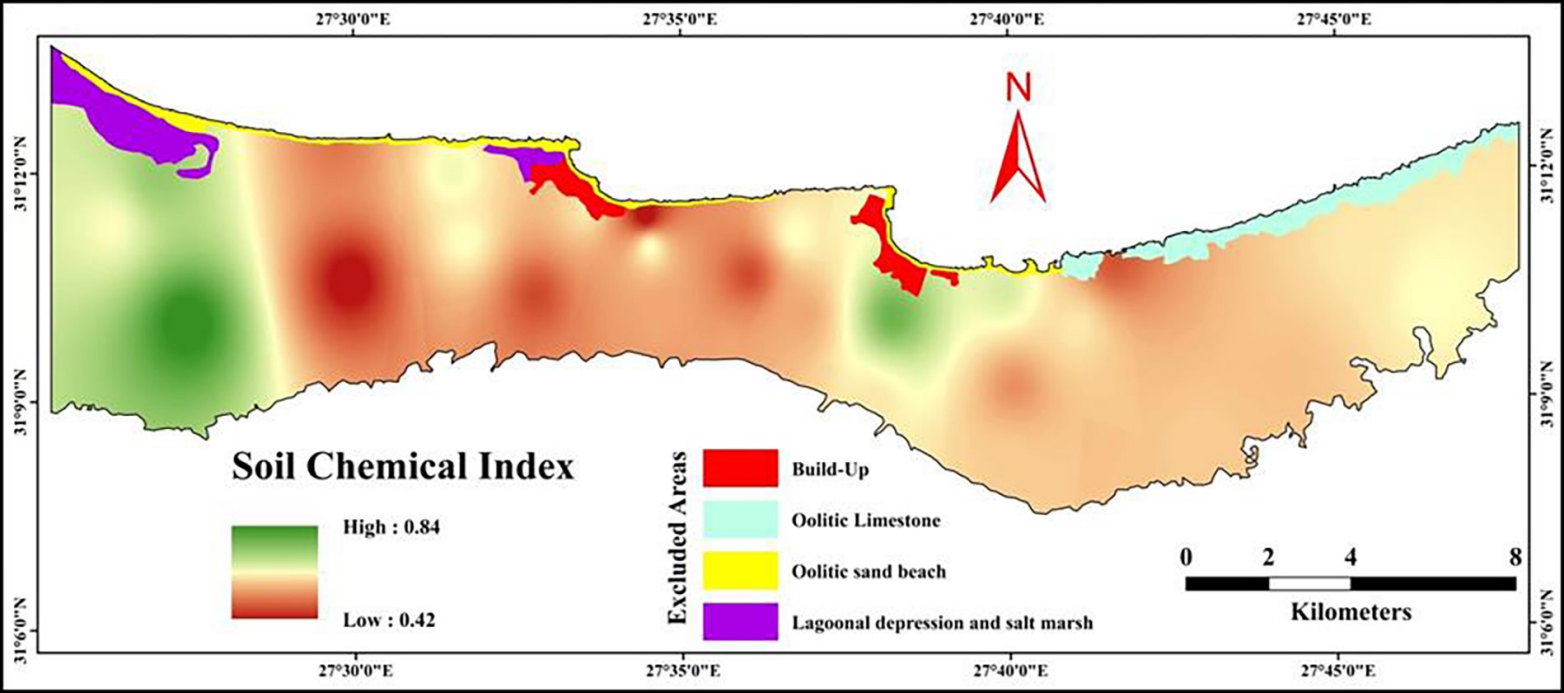
Spatial distribution of soil chemical index.

Data in Table 7 shows the rating of the soil properties used in soil chemical index calculations. Each soil chemical property was rated to have a value between 0.0 and 1.0. The soil organic matter rating value ranged from 0.20 to 0.6 in all soil profiles, while calcium carbonates rated as 1.0 for the whole study area because olives and fig are the most common cultivated trees and they are highly suitable in the calcareous soils with any amount of calcium carbonate [80]. The soil pH rating values varied between 0.60 and 1.00. Regarding the CEC rating, it ranged from 0.20 to 0.6, while the rating of soil EC differed from 0.20 to 1.00. The rating values of ESP varied from 0.60 to 1.0. This rating reflected the status of each soil chemical property in the study area, whereas soil organic matter and CEC had the lowest rating values (staring from 0.20); followed by CaCO_**3**_ (0.40); soil pH and ESP (0.60)21. However, the SCI values ranged from 0.42 to 0.84, as shown in Fig 5. As illustrated in Table 7, the findings showed that the organic matter and cation exchange capacity were the most limited chemical factors in the investigated area, respectively. These characteristics limit soil function since they are essential to plant growth [81]. Hence, choose salt-tolerant crops, apply leaching fraction, and add organic and mineral supplements (like gypsum) are the best recommended practices [24, 82, 83].

**Table 7:** Rating of the soil properties used in calculating quality indicators.

No.	soil chemical index (SCI)						soil physical index (SPI)					soil fertility index (SFI)		
	OM	CaCO_3_	pH	EC	ESP	CEC	Gravel	Texture	BD	WHC	HC	N	P	K
1	0.2	1	1.00	1.00	0.8	0.4	1.00	0.4	0.4	0.6	0.8	0.4	0.2	0.5
2	0.2	1	1.00	0.8	0.8	0.4	1.00	0.7	0.6	1.00	0.6	0.4	0.2	0.5
3	0.4	1	0.8	0.8	0.8	0.4	1.00	0.7	0.6	0.6	1.00	0.4	0.2	0.5
4	0.2	1	0.8	0.6	0.8	0.4	1.00	0.7	0.6	0.8	1.00	0.4	0.2	0.5
5	0.2	1	0.8	0.8	0.8	0.4	1.00	0.7	0.6	1.00	0.6	0.6	0.2	0.5
6	0.2	1	1.00	0.6	0.8	0.4	0.80	0.7	0.6	1.00	1.00	0.4	0.2	0.5
7	0.2	1	0.8	0.8	0.8	0.4	1.00	0.7	0.4	0.8	0.8	0.4	0.2	0.5
8	0.2	1	1.00	0.8	0.8	0.4	1.00	0.7	0.8	1.00	0.6	0.4	0.2	0.5
9	0.4	1	0.8	1.00	1.00	0.6	1.00	0.7	0.6	1.00	0.6	0.6	0.2	0.5
10	0.2	1	0.8	1.00	1.00	0.4	1.00	0.4	0.6	0.8	1.00	0.4	0.2	0.5
11	0.4	1	0.8	0.6	0.8	0.4	1.00	0.7	0.6	1.00	0.6	0.4	0.2	0.5
12	0.6	1	0.8	0.4	0.6	0.6	1.00	0.7	0.6	1.00	0.6	0.6	0.5	0.7
13	0.4	1	0.6	1.00	1.00	0.6	1.00	0.7	0.8	1.00	0.2	0.6	0.2	0.5
14	0.2	1	0.8	0.6	0.8	0.4	1.00	0.7	0.6	0.8	1.00	0.4	0.2	0.5
15	0.2	1	0.8	1.00	1.00	0.4	1.00	0.4	0.4	0.6	0.8	0.4	0.2	0.5
16	0.6	1	1.00	1.00	1.00	0.6	0.80	0.7	0.6	1.00	0.6	0.6	0.2	0.5
17	0.2	1	0.8	1.00	1.00	0.4	1.00	0.7	0.6	1.00	0.6	0.4	0.2	0.5
18	0.2	1	0.6	1.00	1.00	0.2	1.00	0.2	0.4	0.2	0.4	0.4	0.2	0.5
19	0.2	1	1.00	0.8	0.8	0.4	0.80	0.7	0.4	0.6	0.8	0.4	0.5	0.5
20	0.2	1	1.00	0.8	0.8	0.4	0.80	0.7	0.6	0.8	1.00	0.4	0.2	0.5
21	0.2	1	0.6	0.2	0.6	0.4	1.00	0.7	0.6	1.00	1.00	0.4	0.2	0.5
22	0.2	1	0.6	0.2	0.6	0.4	1.00	0.9	0.6	1.00	0.8	0.4	0.2	0.5
23	0.2	1	0.6	0.6	0.8	0.2	1.00	0.4	0.4	0.6	0.8	0.4	0.2	0.5
24	0.2	1	0.6	0.6	0.8	0.2	1.00	0.4	0.4	0.6	0.8	0.4	0.2	0.5
25	0.2	1	0.6	0.8	0.8	0.4	0.80	0.7	0.6	0.8	1.00	0.4	0.2	0.5
26	0.2	1	0.8	0.4	0.8	0.4	0.80	0.7	0.6	1.00	0.6	0.4	0.2	0.5
27	0.2	1	0.6	1	0.6	0.2	1.00	0.2	0.4	0.2	0.2	0.4	0.2	0.5
Min	0.2	1	0.6	0.2	0.6	0.2	0.80	0.2	0.4	0.2	0.2	0.4	0.2	0.5
Max	0.6	1	1.00	1.00	1.00	0.6	1.00	0.7	0.8	1.00	1.00	0.6	0.5	0.7

BD = Bulk Density; WHC = Water Holding Capacity; HC = Hydraulic Conductivity; EC = Electric Conductivity; pH = Soil reaction; CaCO3 = calcium carbonate; OM = Organic Matter; ESP = Exchangeable Sodium Percentage; CEC = Cation Exchange Capacity; N = Nitrogen; K = Potassium; P = Phosphorus

#### 3.5.2 Soil physical index (SPI)

**Fig 6:**
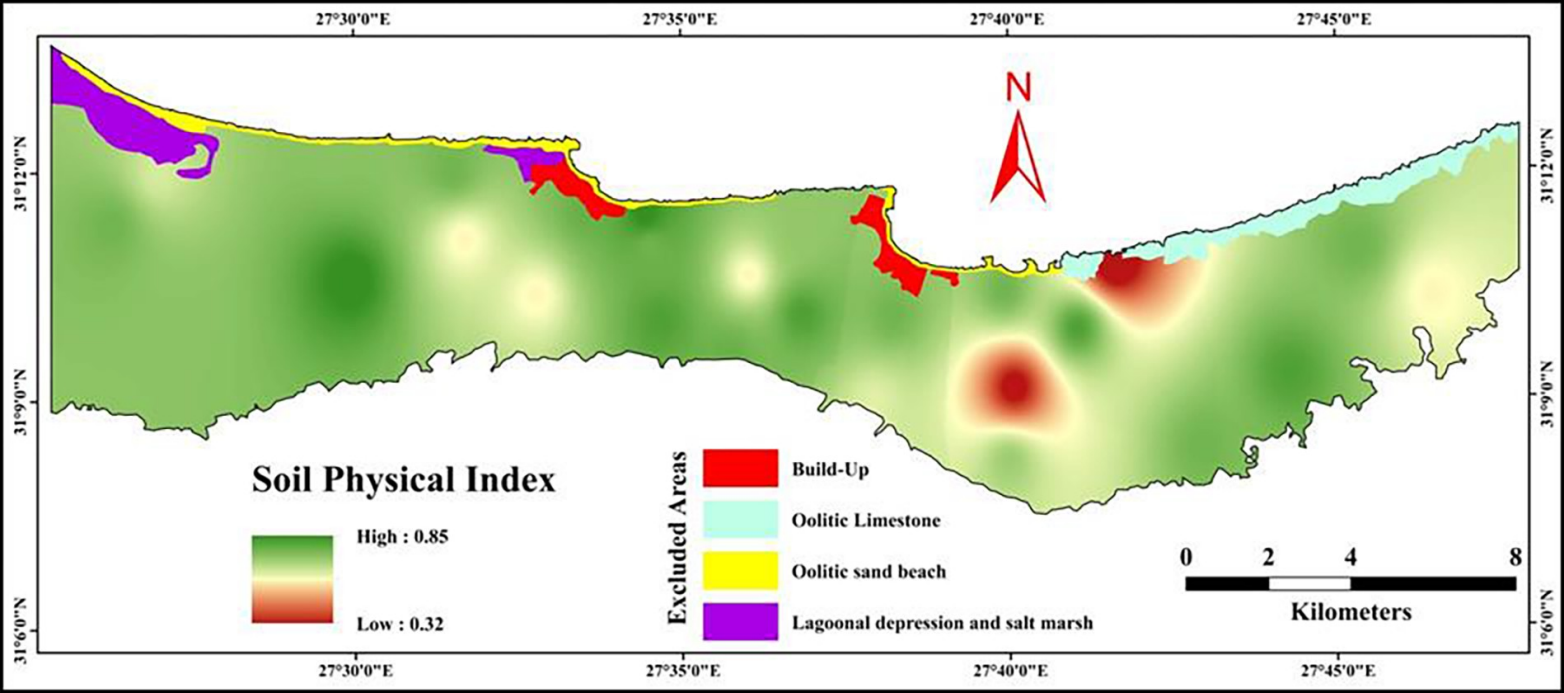
Spatial distribution of soil physical index.

Table 7 reveals the rating values of the tested soil physical properties to calculate the soil physical index. Soil texture rating values varied from 0.20 to 0.70, while hydraulic conductivity ranged from 0.20 to 1.0. Regarding the water holding capacity, it differed between 0.20 to 1.0 while, the bulk density ranged from 0.40 to 0.80 in rating. However, the values of the soil physical index varied between 0.32 and 0.85, as shown in Fig 6. As shown in Table 7, the results showed that the bulk density followed by soil texture was the most limited physical factors in the investigated area. Therefore, improving soil structure through the appropriate use of tillage operations in addition to adding organic and/or mineral amendments are essential to improve the soil physical quality [24, 82, 83].

#### 3.5.3 Soil fertility index (SFI)

**Fig 7:**
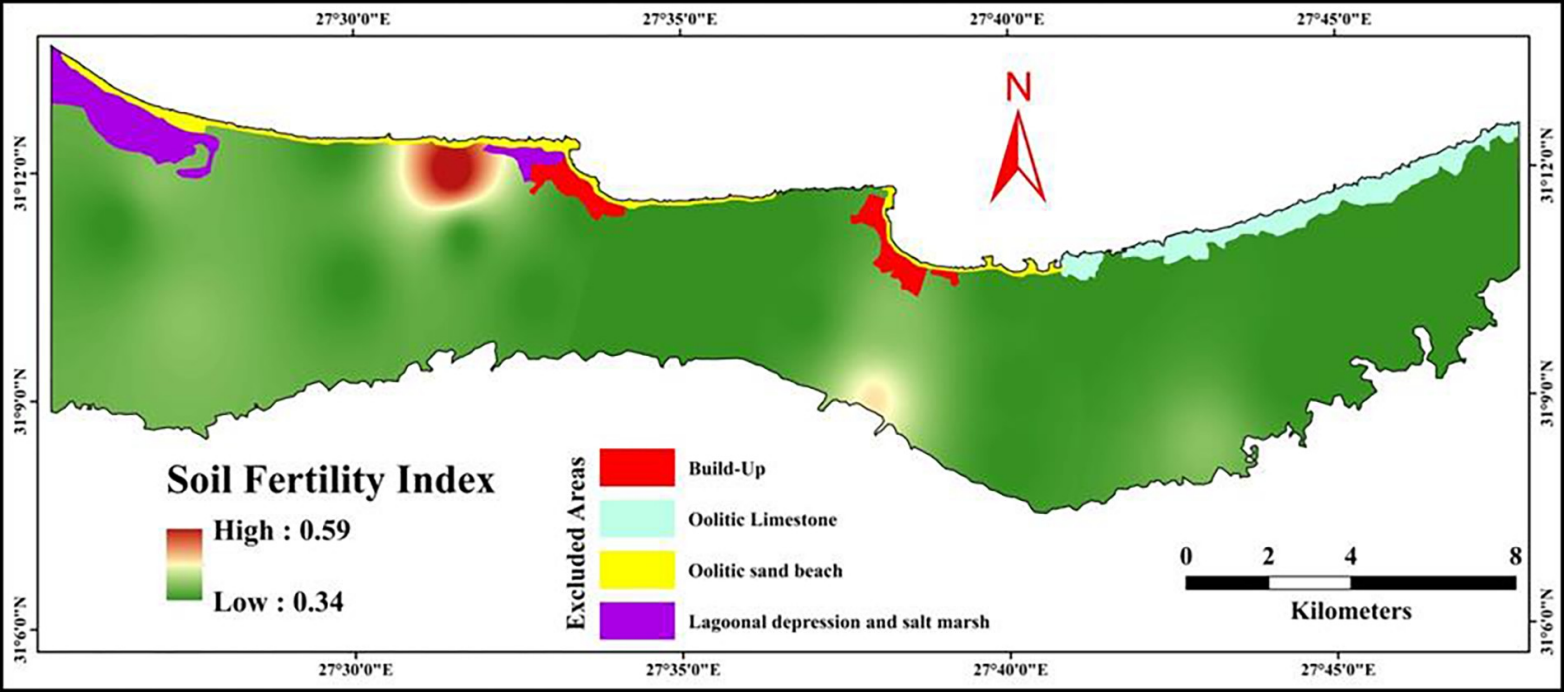
Spatial distribution of soil fertility index.

Table 7 shows the rating values of the soil fertility properties. The available nitrogen rating values ranged from 0.40 to 0.60; available phosphorus varied between 0.20 and 0.50, while available potassium rating values differed from 0.50 to 0.70. However, the values of the soil fertility index varied from 0.34 to 0.59, as shown in Fig 7. As illustrated in Table 7, the findings showed that the available phosphor was the most limited fertility factor in the investigated area. Poor fertility status observed in the studied area resulted from low levels of organic matter and clay contents, which are key components influencing soil fertility [81].

#### 3.5.4 Soil depth

**Fig 8:**
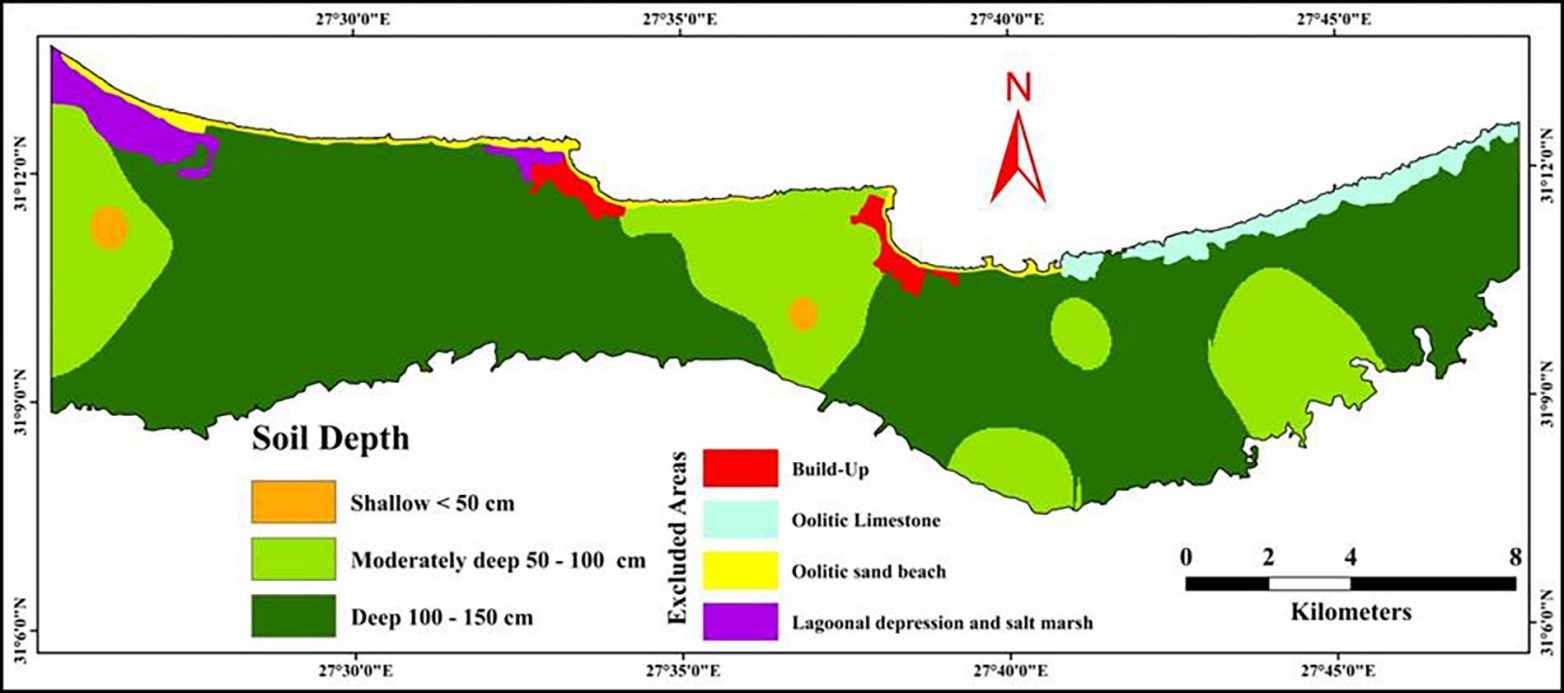
Spatial distribution of soil depth map.

Soil depth plays a critical role in the cultivation of the common trees in the study area such as olive and fig trees, impacting root development, water retention, and nutrient availability. Three categories of soil depth were observed in the investigated area as illustrated in Fig 8 and Table 8. The common soil depth class in the studied area was deep soils with an area of 13353.18 ha representing 67.71% out of the total area. While the moderately deep class covered an area of 4611.03 ha and representing 23.43% out of the total area. On the other hand, very small area was classified as shallow soils with an area of 121.55 ha representing 0.62% out of the total area. Consequently, the rate of the soil depth in the investigated area was ranged between 0.40 and 0.80 as shown in Table 10.

**Table 8:** Distribution of soil depth classes areas in various landforms.

Land form	Shallow		Moderately Deep		Deep	
	Area	%	Area	%	Area	%
LS	121.55	0.62	2635.45	13.36	7222.90	36.62
AF	---	---	497.15	2.52	2110.15	10.70
US	---	---	1403.47	7.12	3773.31	19.13
OLSD	---	---	84.96	0.43	246.82	1.25
Total	121.55	0.62	4611.03	23.43	13353.18	67.71

LS = Lower slope; AF = Alluvial fans; US = Upper slope; OLSD = Oolitic longitudinal sand dunes

#### 3.5.5 Slope gradient

**Fig 9:**
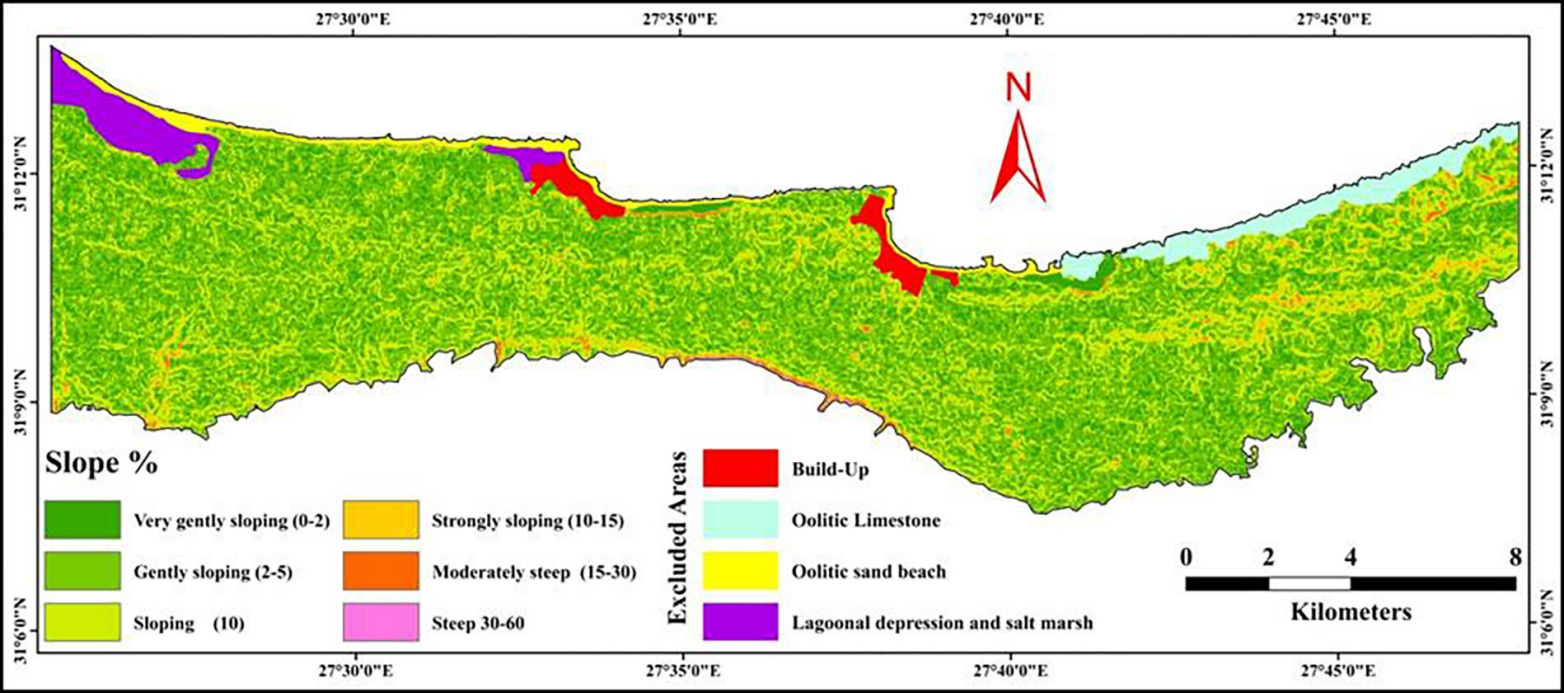
Spatial distribution of slope gradient map.

Three slope classes were observed in the investigated area as shown in Fig 9 and Table 9. These classes were very gently sloping, gently sloping, and sloping covering an area of 3582.61, 9384.75, and 4611.15 ha representing 18.25, 47.58, and 23.30% out of the total area, respectively. As illustrated in Table 10 Very gently sloping class was rated as 0.8, while gently sloping and sloping were rated as 1.0 because the slope gradient from 2 to 10% helps to collect more rain water. Also in theses slope classes the water can infiltrate the soil more effectively and promoting better moisture retention for the cultivated trees. These slopes allow the soil to absorb rainfall without causing too much runoff, which is critical in areas with limited and irregular rainfall, such as the northwestern coast of Egypt. The rate of slope classes more than 10% were decreased due to the rainwater tends to run off quickly before it can be absorbed by the soil.

**Table 9:** Distribution of slope classes areas in various landforms.

Slope class	AF		OLSD		US		LS		Total	
	Area	%	Area	%	Area	%	Area	%	Area	%
Very Gently Sloping	661.70	3.36	111.83	0.57	859.08	4.35	1950.00	9.98	3582.61	18.25
Gently Sloping	1384.79	7.02	119.07	0.60	2580.18	12.68	5300.72	27.28	9384.75	47.58
Sloping	520.69	2.59	89.28	0.39	1500.42	7.18	2500.77	13.14	4611.15	23.30
Strongly Sloping	31.21	0.16	9.10	0.05	180.05	0.91	197.62	1.00	417.98	2.12
Moderately Steep	8.27	0.04	2.50	0.01	47.34	0.24	30.80	0.16	88.91	0.45
Steep	0.64	0.00	0.00	0.00	9.72	0.05	0.00	0.00	10.36	0.05
Total	2607.30	13.17	331.78	1.62	5176.78	25.42	9979.90	51.55	18095.76	91.75

LS = Lower slope; AF = Alluvial fans; US = Upper slope; OLSD = Oolitic longitudinal sand dunes

**Table 10:** Current and potential land productivity indices of the studied soils.

No.	Depth	Slope	SCI	SPI	SFI	CLPI		PLPI	
						Value	class	value	class
1	0.8	1.00	0.54	0.58	0.34	40.47	Good	57.87	Good
2	0.8	1.00	0.52	0.74	0.34	43.27	Good	61.87	Good
3	0.8	1.00	0.57	0.74	0.34	44.41	Good	63.51	Good
4	0.4	1.00	0.51	0.66	0.34	21.44	Poor	30.65	Average
5	0.8	1.00	0.5	0.74	0.39	44.70	Good	61.11	Good
6	0.4	1.00	0.5	0.72	0.34	21.70	Poor	31.03	Average
7	0.6	1.00	0.5	0.66	0.34	31.33	Average	44.80	Good
8	0.6	0.80	0.52	0.77	0.34	26.46	Average	37.84	Average
9	0.8	1.00	0.65	0.74	0.39	48.71	Good	66.58	Good
10	0.6	0.80	0.54	0.7	0.34	25.81	Average	36.91	Average
11	0.8	1.00	0.54	0.74	0.34	43.71	Good	62.50	Good
12	0.8	0.80	0.55	0.77	0.59	42.32	Good	50.33	Good
13	0.8	1.00	0.62	0.64	0.39	45.41	Good	62.08	Good
14	0.8	1.00	0.48	0.77	0.34	42.87	Good	61.30	Good
15	0.8	0.80	0.54	0.63	0.34	32.38	Average	46.30	Good
16	0.8	0.80	0.72	0.77	0.39	39.76	Average	54.35	Good
17	0.4	1.00	0.54	0.66	0.34	21.90	Poor	31.32	Average
18	0.8	1.00	0.46	0.4	0.34	32.47	Average	46.44	Good
19	0.8	1.00	0.56	0.63	0.46	45.26	Good	58.45	Good
20	0.6	1.00	0.56	0.74	0.34	32.59	Average	46.60	Good
21	0.8	0.80	0.36	0.83	0.34	31.72	Average	45.36	Good
22	0.8	0.80	0.39	0.84	0.34	31.78	Average	45.44	Good
23	0.8	1.00	0.44	0.61	0.34	36.79	Average	52.61	Good
24	0.6	1.00	0.41	0.58	0.34	27.59	Average	39.46	Average
25	0.8	1.00	0.51	0.77	0.34	42.24	Good	60.40	Good
26	0.8	1.00	0.45	0.74	0.34	40.51	Good	57.93	Good
27	0.8	1.00	0.42	0.36	0.34	30.14	Average	43.10	Good
Min	0.4	0.80	0.36	0.36	0.34	21.44	Poor	30.65	Average
Max	0.8	1	0.72	0.84	0.59	48.71	Good	66.58	Good

SCI = Soil Chemical Index; SPI = Soil Physical Index; SFI = Soil Fertility Index; CLPI = Current Land Productivity Index; PLPI = Potential Land Productivity Index

### 3.6 Land productivity evaluation

#### 3.6.1 Current land productivity

As we mentioned previously, given the conditions of the study area, the slope gradient and soil depth are considered the most important factors determining the productivity in the study area. Land productivity index was calculated based on SQIs in addition to soil depth and slope gradient. Land productivity classification groups are distinguished in precise numerical units (0–100). Classifications, which meet land productivity requirements, would be taken as the highest grades. Soils with extreme limitations would be the lowest ones. Intermediate grades would be placed in between the two extreme conditions. Values of the factors of land productivity are shown in Table 10. Soil characteristics relevant to land productivity are shown in Table 4, while the rating of these characteristics is shown in Table 7.

**Fig 10:**
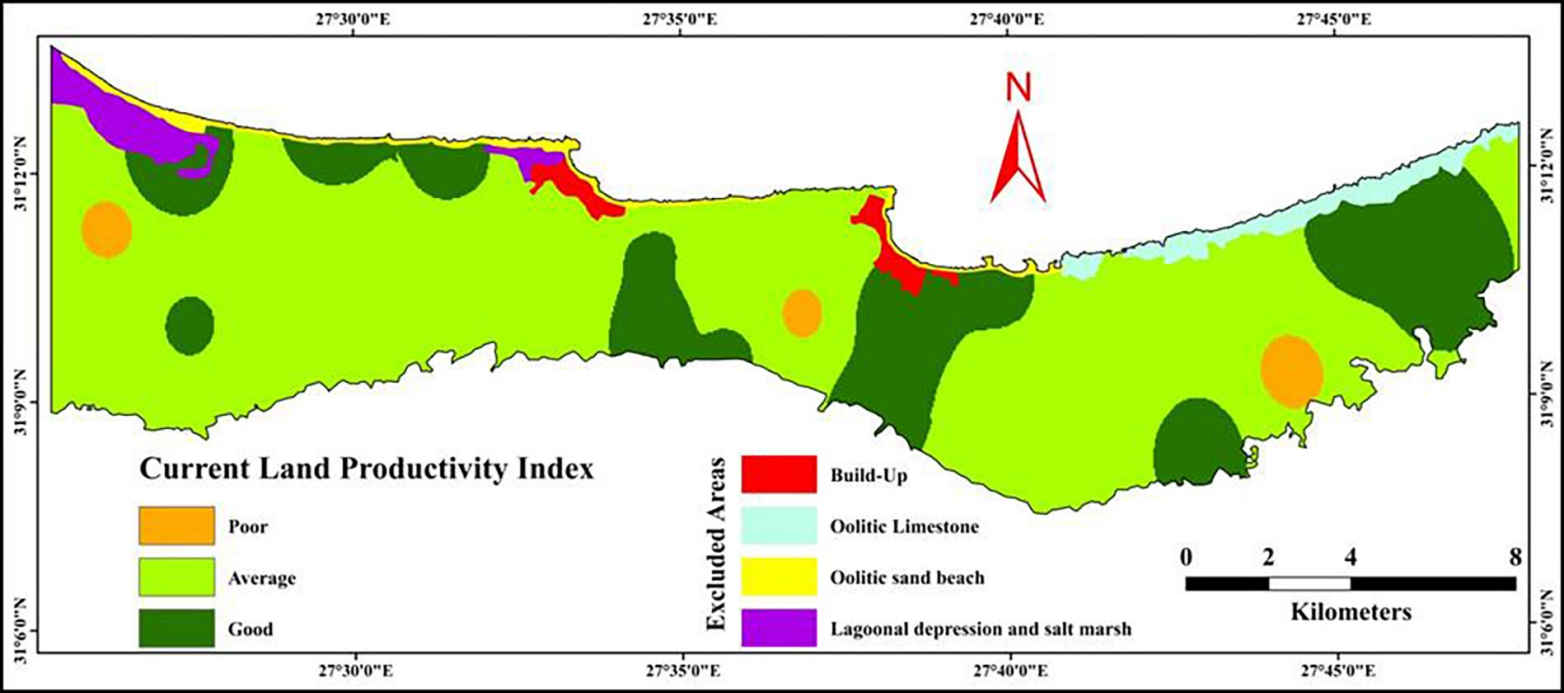
Spatial distribution of the current land productivity index.

The result show that the most common productivity grade in the investigated area is average grade with an area of 13322.63 ha (67.55%) following by good grade with an area of 4339.78 ha (22%). On the other hand, a poor grade covers a very small area (433.35 ha) representing about 2.20% out of the studied area, as illustrated in Fig 10 and Table 11. Mapping unit LS has the productivity grades good, average, and poor with an area of 7434.86 ha (37.70%), 2321.69 ha (11.77%), and 223.35ha (1.13%), respectively. Mapping unit US has the productivity grades average, good, and poor with an area of 3904.12 ha (19.80%), 1062.66 ha (5.39%), and 210.00 ha (1.06%), respectively. Mapping unit AF has the productivity grades good and average with an area of 793.30 (4.02%) and 1814.00 ha (9.20%), respectively. Mapping unit OLSD has the productivity grades good and average with an area of 162.13 (0.82%) and 169.65 ha (0.86%), respectively.

**Table 11:** Areas in hectare of the current land productivity grades.

Land form	Average		Good		Poor	
	Area	%	Area	%	Area	%
US	3904.12	19.80	1062.66	5.39	210.00	1.06
LS	7434.86	37.70	2321.69	11.77	223.35	1.13
AF	1814.00	9.20	793.30	4.02	---	---
OLSD	169.65	0.86	162.13	0.82	---	---
Total	13322.63	67.55	4339.78	22.00	433.35	2.20

LS = Lower slope; AF = Alluvial fans; US = Upper slope; OLSD = Oolitic longitudinal sand dunes

#### 3.6.2 Potential land productivity

**Fig 11:**
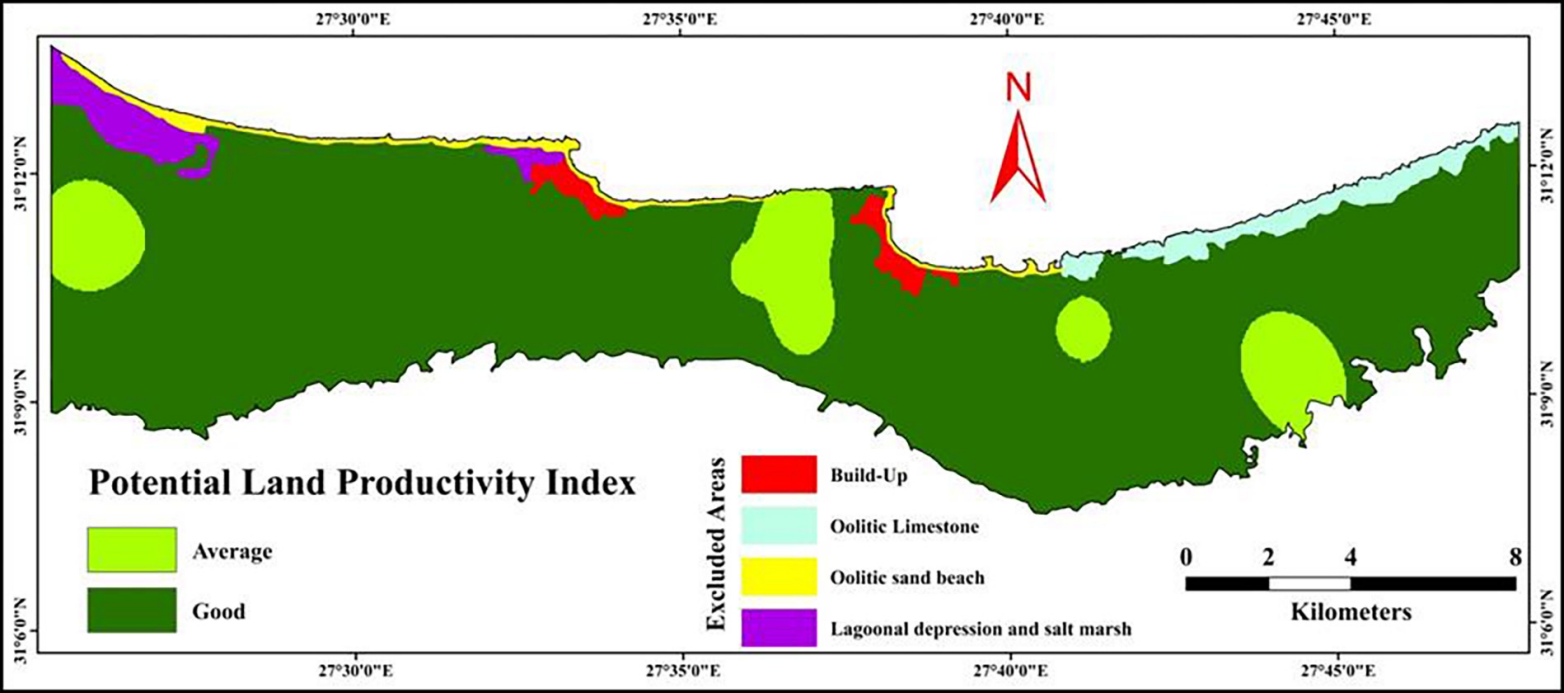
Spatial distribution of potential land productivity index.

To make agricultural policy decisions, accurate forecasts of future soil productivity are required. Limitations of land productivity were derived from the data of current productivity index. Therefore, for estimating potential land productivity, several soil parameters should be modified to increase the land productivity of the research region. The most limiting factor for land productivity in the investigated area was soil fertility index (N, P, and K). For improving soil fertility, addition of N, P, and K fertilizers should be applied continuously in the study area. In order to calculate PLPI, the rating of N, P, and K were increased to 1.0. PLPI after improvement is shown in Table 10. From the future or potential productivity index data of the study area, it was clear that the land productivity was increased after the proposed solutions of soil improvement. The results show that most of the study area consists of good (class II) land productivity covering an area of 16192.54 ha representing 82.10% out of the total area. On the other hand a smaller area of 1903.23 ha (9.65% of the total area) is of an average productivity (class III), as illustrated in Fig 11 and Table 12. The good grade of land productivity is distributed among the land form units US, LS, AF, and OLSD with an area of 4660.82, 8714.18, 2523.59, and 293.95 ha representing 23.63, 44.19, 12.80, and 1.49% out of the investigated area, respectively.

**Table 12:** Areas in hectare of potential land productivity index.

Landform	Good		Average	
	Area	%	Area	%
US	4660.82	23.63	515.96	2.62
LS	8714.18	44.19	1265.72	6.42
AF	2523.59	12.80	83.72	0.42
OLSD	293.95	1.49	37.83	0.19
Total	16192.54	82.10	1903.23	9.65

LS = Lower slope; AF = Alluvial fans; US = Upper slope; OLSD = Oolitic longitudinal sand dunes

#### 3.6.3 Change in the land productivity

As shown in Table 13, the productivity class “Average” was decreased from 13322.63 ha to 1903.23 ha with a total change of—57.94%. On the other hand, the productivity class “Good” was increased from 4339.78 ha to 16192.54 ha with a total change of + 60.14%. The finding showed that more than 60% of the studied area was converted from poor and average class to good class after applying the proposed solutions of soil improvement.

**Table 13:** Areas in hectare of actual and potential productivity classes in study area.

Productivity class			CLPI (before improvement)		PLPI (after improvement)		Change %
Index range	Definition	Symbol	Area (ha)	%	Area (ha)	%	
40–70	Good	II	4339.78	22.00	16192.54	82.10	+ 60.14
25–40	Average	III	13322.63	67.55	1903.23	9.65	- 57.94
10–25	Poor	IV	433.35	2.20	---	---	- 2.20

CLPI = Current Land Productivity Index; PLPI = Potential Land Productivity Index

## 4. Discussion

### 4.1 Soil quality indicators

The results of the soil chemical index (SCI) assessment provide critical insights into the chemical properties of soils in the northwestern coast of Egypt. This region, known for its arid conditions, often faces challenges related to soil fertility and land productivity. Understanding the behavior of various soil chemical properties can help guide appropriate management interventions for sustainable agricultural production. The findings showed that the organic matter and cation exchange capacity were the most limited chemical factors in the investigated area. These characteristics limit soil function since they are essential to plant growth [81]. Hence, choose salt-tolerant crops, apply leaching fraction, and add organic and mineral supplements (like gypsum) are the best recommended practices [24, 82, 83].The soil physical index (SPI) was calculated using soil texture, hydraulic conductivity, water holding capacity, and bulk density. Texture ratings varied from 0.20 to 0.70, reflecting a range of soil textures from sandy to loamy. Sandy soils, which likely account for the lower ratings, have poor water and nutrient retention capabilities, making them less suitable for agriculture without interventions like organic matter addition or mulching [84]. As demonstrated by the results, the most constrained physical parameters in the studied area are bulk density and soil texture along the same line as [85, 86]. Therefore, improving soil structure through the appropriate use of tillage operations in addition to adding organic and/or mineral amendments are essential to improve the soil physical quality [82]. The soil fertility index (SFI) was based on the availability of nitrogen, phosphorus, and potassium. The overall SFI values, ranging from 0.34 to 0.59, indicate that fertility is a limiting factor in the study area. The findings showed that the available phosphorus is the most limited fertility factor in the investigated area. Poor fertility status observed in the studied area resulted from low levels of organic matter and clay contents, which are key components influencing soil fertility [81]. Targeted fertilizer application, especially phosphorus, will be necessary to improve soil fertility and boost crop yields. Maintaining soil fertility and raising the SOM level in the study area can be achieved by growing a variety of seasonal crops rather than replanting the same ones in the same locations [87–91]. This contributes to the long-term preservation of soil quality, which raises crop productivity [92].

Soil depth plays a critical role in the cultivation of the common trees in the study area such as olive and fig trees, impacting root development, water retention, and nutrient availability. The study area is characterized predominantly by deep soils, covering 67.71% of the total area. The presence of deep and moderately deep soils provides favorable conditions for agriculture, particularly for tree crops like olives and figs, which require well-drained soils with good root penetration [37]. However, shallow soils, though limited in extent, may pose a challenge for cultivation, requiring careful management to avoid erosion and nutrient depletion. The study area is predominantly classified as gently sloping, covering 47.58% of the total area. Sloping areas help in rainwater infiltration and prevent excessive runoff, which is crucial in arid regions where water is a limiting resource [93]. Gently sloping and sloping areas (23.30%) are rated highly for their ability to retain and absorb rainwater, making them suitable for rain-fed agriculture. The slope gradient is particularly important for rain-fed cultivation in the northwestern coast of Egypt, where water availability is limited and irrigation is not typically used [49, 94, 95]. In this context, managing the slope becomes crucial for optimizing water retention, minimizing soil erosion, and maintaining soil fertility. The rate of slope classes more than 10% were decreased due to the rainwater tends to run off quickly before it can be absorbed by the soil. These results are consistent with many studies [48, 96]. Overall, the slope gradient in the study area supports rainwater infiltration, reducing the risk of erosion and promoting moisture retention, which is essential for the cultivation of trees in this dry region. Overall, the analysis of soil quality indicators reveals both challenges and opportunities for soil management in the northwestern coast of Egypt. While the area benefits from deep soils and favorable slope gradients for rainwater absorption, issues such as low organic matter, limited nutrient availability, and varying water retention capacities present challenges for sustainable agriculture. Improving soil chemical and physical properties through targeted interventions, such as organic amendments, irrigation management, and fertilizer application, will be crucial in enhancing soil quality and land productivity. These efforts will be essential in optimizing land use and supporting sustainable agriculture in this arid region.

### 4.2 Land productivity evaluation

The evaluation of current and potential land productivity in the northwestern coast of Egypt offers significant insights into the state of agricultural land and the impacts of targeted interventions on land productivity. The land productivity index (LPI), calculated using soil quality indicators (SQIs) alongside soil depth and slope gradient, reveals the current limitations and potential for improvement in the region.

#### 4.2.1 Current land productivity

The current land productivity (CLPI) in the study area is dominated by average productivity (67.55% of the total area) and good productivity (22%). On the other hand, the poor productivity land covers 2.20% of the total area. These findings suggest that, while the majority of the land is moderately productive, there is substantial room for improvement in soil management practices. The variation in land productivity grades across different mapping units (LS, US, AF, and OLSD) demonstrates that soil properties vary across the landscape, influencing agricultural potential. Mapping unit LS, the largest in terms of area, contains good, average, and poor productivity classes. This suggests a heterogeneous distribution of soil qualities, likely due to differences in soil depth, texture, and chemical properties. Similarly, mapping unit US also contains a mix of productivity classes, although it leans more toward average and good grades. Units AF and OLSD predominantly contain good and average productivity soils, which are favorable for agricultural development. The factors limiting land productivity, particularly in areas classified as average or poor, are likely linked to soil fertility, texture, and structure. For example, low levels of essential nutrients (nitrogen, phosphorus, potassium) and poor soil physical properties such as low water retention may restrict plant growth and yield in these areas.

#### 4.2.2 Potential land productivity

After implementing soil improvement strategies, the potential land productivity (PLPI) in the study area increased significantly. The most limiting factor for land productivity—soil fertility—was addressed through the continuous application of nitrogen, phosphorus, and potassium fertilizers. Following these improvements, the good productivity class (Class II) increased to 82.10% of the total area, while the average class (Class III) was reduced to 9.65%. This significant shift in productivity highlights the effectiveness of soil management interventions in enhancing agricultural productivity potential. The distribution of good land productivity across the mapping units reflects how targeted soil improvement strategies can maximize land use. The largest gains in productivity were seen in mapping unit LS, which saw a significant increase in good productivity from 37.70% to 44.19% of its total area. This indicates that, with appropriate management, even areas with initially average or poor productivity can be substantially improved, transforming previously marginal lands into more productive agricultural areas.

#### 4.2.3 Change in land productivity

The data on changes in land productivity underscore the transformative effect of soil improvement measures. The "average" productivity class was reduced by 57.94%, while the "good" productivity class increased by 60.14%. This remarkable change indicates that more than 60% of the studied area transitioned from poor or average productivity to good productivity, reinforcing the critical role that soil fertility management plays in enhancing potential agricultural productivity potential. These results highlight the importance of a targeted approach to soil management in arid regions like the northwestern coast of Egypt. Soil depth and slope gradient were identified as key factors influencing land productivity, particularly in this area, where rainwater infiltration and retention are crucial for crop success. The slopes in this region, which encourage rainwater collection and absorption, coupled with deep soils, provide an ideal foundation for agriculture, particularly when combined with soil fertility enhancements.

### 4.3 Implications for agricultural development

The findings from this study have significant implications for agricultural development and land use planning in the studied region. The dramatic improvement in land productivity after soil fertility enhancement highlights the potential for transforming marginal lands into productive agricultural zones, which is crucial for addressing food security challenges in Egypt’s arid regions. The study also underscores the importance of soil fertility management, particularly through the continuous application of essential nutrients like nitrogen, phosphorus, and potassium. Furthermore, this approach to assessing and improving land productivity can be replicated in similar arid and semi-arid regions. The integration of soil quality indicators with slope and depth evaluations provides a strong framework for understanding land productivity and guiding sustainable agricultural practices. In arid regions, where water and soil fertility are often limiting factors, such targeted interventions can help maximize productivity while ensuring the long-term sustainability of natural resources. In general, the study demonstrates that significant improvements in land productivity can be achieved through strategic soil management interventions. By addressing key limitations in soil fertility, the region’s agricultural potential has been greatly enhanced, contributing to improved land use efficiency and food security. These findings offer valuable insights for policymakers, land managers, and farmers in the region, providing a clear path toward more productive and sustainable agricultural practices.

## 5. Conclusions

Precisely assessing of land productivity is a very crucial issue for precision farming (in particular) and for the appropriate management of sustainable agricultural methods (in general). This assessment facilitates the identification of the most appropriate crops and the possible agricultural uses of the area. Land productivity is affected by agricultural practices and climatic conditions, which, in turn, affect the physical, chemical, and fertility properties of the soil. In this study, the nutrients and physical and chemical properties of the soil were used to assess LPI based on SQIs in some areas of northwestern coast of Egypt. In order to accomplish this investigation, land productivity equation was jointly used with spatial modeling in GIS to obtain, calculate, and map CLPI and PLPI of the study area. The results of SQIs showed that, the most dominant limiting factors were OM, N, P, K, CEC, N, BD, and soil texture. The results of current land productivity index (CLPI) showed that the most common productivity grades in the investigated area were average and good grads covering an area of 13322.63 ha (67.55%) and 4339.78 ha (22%). The most limiting factor for land productivity in the investigated area was soil fertility index (N, P, and K). For improving soil fertility, addition of N, P, and K fertilizers should be applied continuously in the study area. On the other hand, the results of potential land productivity index (PLPI) after soil fertility improvements showed that most of the study area (16192.54 ha; 82.10% of area) consists of good (class II) land productivity. While a smaller area of 1903.23 ha (9.65% of the total area) is of an average productivity (class III). Results showed that the change in the “Average” land productivity grade was decreased from 13322.63 ha to 1903.23 ha with a total change of—57.94%. %. On the other hand, the productivity class “Good” was increased from 4339.78 ha to 16192.54 ha with a total change of + 60.14%. The finding showed that more than 60% of the studied area was converted from poor and average class to good class after applying the proposed solutions of soil improvement. As a whole, the results reflect that the joint use of spatial modeling and GIS allows for an accurate and effective assessment of CLPI and PLPI. Improving soil productivity requires good land management including addition mineral fertilizers (N, P, and K) and addition of organic matter to improve soil structure. In conclusion, by incorporating soil physical, chemical, and fertility indicators, the spatial model put out in this work may provide a more precise methodology for evaluating the spatial distribution of land productivity in similar conditions.
